# P020 Too close to the bone - Paget’s disease

**DOI:** 10.1093/rap/rkag113.050

**Published:** 2026-11-03

**Authors:** Catherine McGrath

**Affiliations:** Frimley Park Health NHS Foundation Trust, Frimley, United Kingdom

## Abstract

**Introduction:**

Paget’s disease of bone (PDB) is the second most common metabolic bone disease after osteoporosis. It is characterised by increased but disorganised bone remodelling which can result in deformity, bone fragility and fracture. It has a strong genetic element, with a family history being noted in 10–20% of cases. Suspicion for PDB may be raised by increased alkaline phosphatase or incidental findings on imaging.

**Case description:**

A 75 y.o. woman, a retired radiographer, was seen in clinic for management of her psoriatic arthritis treated with methotrexate. She complained of progressive low back pain and plain X-ray imaging of her lumbar spine and pelvis was undertaken. The initial report described lytic change and expansion of the L2 lumbar vertebrae with coarsened trabeculation in the left superior and inferior pubic rami. The differential diagnosis included potential metastasis, myeloma, or lymphoma and Paget’s disease. Alkaline phosphatase at the time was in the normal range.

Our patient took great interest in the radiology findings and was understandably concerned as to the possibilities. She disclosed that her mother and cousin had both been diagnosed with PDB. MRI imaging of the affected areas confirmed widening and loss of height of the L2 vertebral body consistent with PDB. No other diagnosis was thought to fit. Pubic rami appearance on MRI did not confirm PDB. Further nuclear medicine bone imaging described low grade increased uptake seen in the left pubic bone and in the L2 vertebral body. DEXA bone density scanning showed bone density in the osteopenia range in the lumbar spine with the lowest density at the L2 vertebra. Due to ongoing lower back pain symptoms, a single infusion of zoledronic acid was administered and she reported improved pain scores.

Throughout this time, she continued treatment for her psoriatic arthritis and accumulated other new diagnoses each with their own treatment plans. She was treated with anti-tuberculous drugs for latent tuberculosis infection. She developed neutropenia and eventually managed to come off the disease modifying drugs for her psoriatic arthritis. More recently, her alkaline phosphatase has started to rise for the first time and it is unclear whether this is further manifestation of her PDB and further investigations are underway.

**Discussion:**

This case shows that PDB can be picked up as an incidental finding on imaging but the appearance can be unexpected and mimic other conditions causing concern to patient and physician alike. The most common sites affected are pelvis, spine and skull. Up to 90% of cases of PDB are asymptomatic with or without raised alkaline phosphatase. The bisphosphonate zoledronic acid can help slow down bone resorption, stabilize the disease, and help manage pain. It is still unknown whether bisphosphonate treatment prevents long term complications of the disease. Not all cases require intervention however and treating raised alkaline phosphatase due to PDB in the absence of symptoms in not recommended.

The UK has one of the highest rates of Paget’s disease globally but the overall incidence has dropped significantly in the last 25 years with currently around 1 in 50,000 persons affected. Some areas of the country are still regarded as relative hot spots especially North West England and in areas of high socioeconomic deprivation.

PDB is not simply a skeletal disease but other problems can be caused by the hyperactive bone remodelling process. This can include the cardiovascular system (high output heart failure), metabolic system (hypercalcaemia) and can, in rare cases, progress to osteosarcoma. Hearing loss, headaches, sciatica, and balance issues may also be seen.

In people with a family history of Paget’s disease, testing for pathogenic variants in the SQSTM1 gene has been used to detect people with early asymptomatic disease. In these people, prophylactic treatment with zoledronic acid can modify disease progression.

**Key learning points:**

1. PDB may have become a less common metabolic bone condition but it is still out there

2. The condition may be an incidental finding and the patient asymptomatic

3. Treating symptomatic cases with zoledronate may improve pain but may not reduce fracture risk

4. Family history is important in this condition and genetic testing has a role to play

